# Modified Methylation Following Electrostimulation in a Standardized Setting—Complementing a Transcriptomic Analysis

**DOI:** 10.3390/cells14110838

**Published:** 2025-06-04

**Authors:** Biagio Di Pietro, Simona Villata, Anna Plaksienko, Tiziana Guarnieri, Simeone Dal Monego, Margherita Degasperi, Pietro Di Lena, Danilo Licastro, Claudia Angelini, Francesca Frascella, Lucia Napione, Christine Nardini

**Affiliations:** 1 Consiglio Nazionale delle Ricerche, Istituto per le Applicazioni del Calcolo “Mauro Picone”, 00185 Roma, Italy; dipietro@iac.cnr.it (B.D.P.); anna.plaksienko@medisin.uio.no (A.P.); tiziana.guarnieri@unibo.it (T.G.); claudia.angelini@cnr.it (C.A.); 2 Dipartimento di Scienza Applicata e Tecnologia, Politecnico di Torino, 10129 Turin, Italy; simona.villata@polito.it (S.V.); francesca.frascella@polito.it (F.F.); 3 PolitoBIOMed Lab, Politecnico di Torino, 10129 Turin, Italy; 4 Oslo Center of Biostatistics and Epidemiology, University of Oslo, 0317 Oslo, Norway; 5 Dipartimento di Scienze Biologiche, Geologiche e Ambientali (BIGEA), University of Bologna, 40100 Bologna, Italy; 6 Area Science Park, Basovizza, 34149 Trieste, Italy; simeone.dalmonego@areasciencepark.it (S.D.M.); margherita.degasperi@areasciencepark.it (M.D.); danilo.licastro@areasciencepark.it (D.L.); 7 Department of Computer Science and Engineering, University of Bologna, 40100 Bologna, Italy; pietro.dilena@unibo.it

**Keywords:** inflammation, electrostimulation, 3D bioconstruct, methylation, methylage

## Abstract

Electrical stimulation (ES) is widely employed in both clinical therapies and research settings where it has shown promise in promoting tissue regeneration, wound healing, and inflammation control. Research has also highlighted ES as a regulator of DNA demethylation, which plays a critical role in nerve regeneration and cellular repair mechanisms. While the impact of ES on epigenetic processes is recognized, its broader effects on cellular functions, particularly in inflammation and wound healing, are less understood. We recently showed how ES impacts inflammatory states by modulating transcriptomic and metabolomic profiles in a 3Din vitromodel where human fibroblasts and keratinocytes are included in a collagen matrix, i.e., even in the absence of the nervous system. Here, we propose to deepen our exploration on the differential effects on DNA methylation, including an investigation of the correlation with age acceleration using a mitotic clock. These results confirm and caution on the differential effect of DC on inflamed and non-inflamed samples and suggest an involvement of direct current stimuli at 1 V (DC1) in the control of senescent processes associated with mitosis and inflammation; the mechanistic details of these will have to be clarified with additional experiments.

## 1. Introduction

Electrical stimulation (ES) has demonstrated a wide range of effects on various biological processes, including galvanotaxis and cell proliferation [1]. Additionally, it exhibits anti-inflammatory properties through the actions of specialized cells in the autonomic nervous system, such as in bioelectronic medicine and vagus nerve stimulation (VNS), which depend on the inflammatory reflex [2,3].

Based on this, it is clear that ES anti-inflammatory applications hold great translational potential, given the ubiquitous role of inflammation in the silent subclinical inflammatory phase that precedes non-communicable diseases (NCDs), which can cause pandemics [4] and mobilize worldwide efforts (Sustainable Development Goals, SGD 3.4).

Two major challenges are faced when proceeding with actual medical translation, namely the generation of a continuum between basic research findings and medical applications (mechanistic insights into the effectiveness of ES) and the standardization of experimental settings enabling meta-analyses.

To contribute directly to solving both challenges, we recently proposed to investigate the impact of electrical stimuli on inflammation and wound healing within a three-dimensional bioconstructed sample, which is highly reproducible, for the systematic observation of ES effects in different contexts [5]. This recent work has shown how, at the transcriptional–metabolomic level, ES effects are dependent on the surrounding microenvironment, whether physiological or inflamed; on the nature of the stimulus; and, of course, on time. Notably, in our experimental setting, direct current (DC) stimuli tend to have a more pronounced effect compared to alternate current (AC) ones. In particular, DC at 1 V shows moderate pro-inflammatory activity in a physiological context, while DC at 5 V exhibits, in our setting, anti-inflammatory properties. Furthermore, AC at 10 Hz and 5 V is observed to promote proliferation in inflamed states, with an inverse effect under physiological conditions. Finally, we observed senescence-like events, first as a natural process occurring over time (i.e., in the absence of stimuli) and faster in inflamed samples and second under DC at 1 V and AC at 10 Hz in physiological samples.

Building upon these insights, the present study explores the epigenomic dimension, focusing on DNA methylation. Our objective is to examine whether different types of electrical stimuli can modulate DNA methylation patterns in such a short time frame (48 h). Furthermore, given the long established ability of selected methylation signals to correlate with biological aging (methylage) [6,7], we exploit the cellular epigenetic mitotic clock EpiTOC [8] for this additional analysis.

Clearly, relevant and promising observations derived from this *omic* analysis will require further validation with low-throughput experiments guided, advantageously, by the present results. A scheme representing the whole analysis is given in Figure S1 of the Supplementary Materials.

## 2. Materials and Methods

### 2.1. Study Design

The experimental setup is described in detail in [5]. Briefly, we constructed a 3D in vitro model composed of a collagen matrix embedded with human fibroblasts (HFF-1 cell line, ATCC) overlaid with human keratinocytes (HaCaT cell line, Antibody Research Corporation, St. Charles, MO, USA), following the protocol described in the Supplementary Materials. The initial (cell seeding day) ratio of fibroblasts to keratinocytes was equal to approximately 3.75 (7.5 × 10^5^ HFF-1 to 2 × 10^5^ HaCaT). A proxy of the ratio between the two cell types at the end of the maturation can be estimated from the doubling time in 2D culture: for fibroblasts, it lies in the range of 24–36 h; for keratinocytes, it is in 18–24 h. However, given the time frame for model maturation (14 days), it has to be considered that both cell lines may have variations in their doubling time due to the environment, the change from 2D to 3D culture [9], and the crosstalk between the two cellular components [10,11]. The 3D in vitro models were stimulated with four different types of ES (1 and 5 V in DC and 10 and 100 Hz at 5 V in AC) sampled at three time points (baseline, labeled 0, and then 1 and 48 h post-stimulation). Inflammation (INFL) was mimicked by administration of TNF-α prior to electrostimulation, and non-INFL samples were labeled physiologic (PHYS). The study design is reported in Table 1. All samples are intuitively named after the labels described.

### 2.2. Methylomics

DNA was extracted from the entire 3D in vitro skin model, containing both fibroblasts and keratinocytes, using Quick-DNA/RNA™Microprep Plus Kit from Zymo Research (Irvine, CA, USA), stored at −80 °C and further processed by bisulfite treatment with Zymo EZ DNA Methylation Kit (Zymo Research, Irvine, CA, USA) to convert unmethylated cytosines to uracils, according to manufacturer’s instructions. DNA methylation was assessed via Infinium MethylationEPIC v1.0 BeadChip Kit (Illumina, San Diego, CA, USA), following the producer’s indications. The iScan System (Illumina, San Diego, CA, USA) was used to read the BeadChips and methylation data were obtained in the form of intensity data (IDAT) files and processed using the ChAMP package in R [12]. Data were stored and are available on GEO with ID GSE280243.

### 2.3. Differential Analysis

Following data import, quality control was performed using multidimensional scaling (MDS) and density plots to assess data distribution and identify outliers. Normalization with beta-mixture quantile dilation (BMIQ) and batch effect correction (ComBat, accounting for “Run” and “Array”), were checked through singular value decomposition (SVD) and principal component analysis (PCA) plots. After *logit*-transforming the batch-corrected counts, the interactions between the state, time, and stimulus were linearly modeled, and differential analysis was run using the limma package [13]. Differentially methylated probes (DMPs) were finally identified using the topTable function.

### 2.4. Enrichment

To gain insights into the biological pathways and processes associated with the DMPs, enrichment was computed with the function gometh from the missMethyl package [14] for the GO *molecular functions* categories. This approach allows us to exploit the molecular functions associated with transcripts to CpGs, since CpG sites are mapped to Entrez Gene IDs, and enrichment is computed taking into account both the number of CpG sites per gene on the EPIC array and the CpGs annotated for multiple genes. Results are presented as dotplots where the intensity of the color corresponds to the quantity of differentially expressed genes (DEGs) and the radius is inversely proportional to the corrected q-value (FDR q-value Bonferroni-corrected for the total number of contrasts). Enrichment is run contrast-wise; however, for the sake of readability, discussion and comparison to previous work [5] are performed with constrasts grouped under six main questions (see Table 2).

### 2.5. Methylage

The EpiTOC model [8] calculates a statistical score (*pcgtAge*) that significantly correlates with chronological age in healthy tissue samples that also approximates a mitotic-like clock in cell lines. Hence, *methylage* and *mitotic age* are used in this context interchangeably, despite the mitotic clock computing a more specific marker than epigenetic clocks. *pcgtAge* is computed as the *Z*-score of the batch-corrected normalized β values of 385 CpGs (largely selected from the polycomb target genes) in the two conditions to be compared. In our case, all fifty-seven contrasts were processed with an unpaired *t*-test, according to the number of replicas in each population. A correction for multiple hypotheses was computed with a diversity of approaches using the mt.rawp2adjp function from the R package multtest. A selection and the full list of contrasts; Δm, which indicates the difference in the average methylation; t-statistics; *p*-values; and corrections are reported in Table 3 and Table S1, respectively.

## 3. Results and Discussion

In this section, to guide our exploration across the numerous samples available, we explore six questions, listed in the first column of Table 2 via the enrichment results of the corresponding fifty-seven contrasts shown in the second column. To ease the discussion, the third column recalls the joint effects on transcriptomics and metabolomics discussed in [5], and finally, the fourth column summarizes the discussion on methylomics that will be presented below. All results are discussed based on enrichment analysis (see Section 2) and refer therefore to the differential biological activity that emerges from statistically significant variations of the associated genes and not to quantitative alterations in defined marker proteins. In particular, enrichment is presented question-wise with a bubble plot indicating enrichment of the contrasts (on the x-axis) for the gene sets on the y-axis. The challenge we face lies in the large number of contrasts, given the complex experimental design.

Finally, EpiTOC-relevant results are discussed here with the support of Table 3, with the complete results of the analysis available in Table S1. The selection focuses on the contrasts that present with a stable (late, 48 h) (i) association with senescence-like events in transcriptomics and (ii) a statistically significant association with accelerated mitosis (before correction).

The first group includes the effect of inflammation on unstimulated samples and of DC1 and AC10 on physiological samples. While the first association with senescence-like events is confirmed (INFLvsPHYS.48.NO) and even survives correction for multiple hypotheses, the two latter (PHYS.48.DC1vsNO and PHYS.48.AC10vsNO) events are not associated with enhanced mitosis. However, DC5, associated in transcriptomics with *transitional* activity, presents signs of accelerated mitosis (PHYS.48.DC5vsNO). This is in line with the training and validation of EpiTOC, which appears to be robustly associated with accelerated mitosis in cancer, including in pre-cancerous lesions.

The last two contrasts refer to the effects of DC1. In transcriptomics, DC1’s effect on INFL is associated with proliferation, while on PHYS it is associated with senescent-like activity. Interestingly, when comparing the differential effect on PHYS and INFL samples, i.e., the first and last rows of Table 3, we observe a negative variation in the average methylation (Δm) of the clock CpGs at DC1 (INFL.48.DC1vsNO), that is, the opposite of what we observe in unstimulated conditions (INFLvsPHYS.48.NO). This implies that the senescence-like effect (including reduced proliferation) observed in aging PHYS at the transcriptional level (48 h) is associated at the methylation level with variations that are stronger than the senescent-like effect that accompanies aging INFL, with an overall negative balance.

In other words, the enhanced proliferation elicited by DC1 on inflamed samples appears to compensate for the reduced proliferation that accompanies inflammation, possibly resetting the mitotic clock to a more physiologic amount of ticks.

Certainly, more investigation is warranted in this direction to elucidate the potential relevance of these observations.

### 3.1. Question 1: Impact of Time

**Figure 1:**
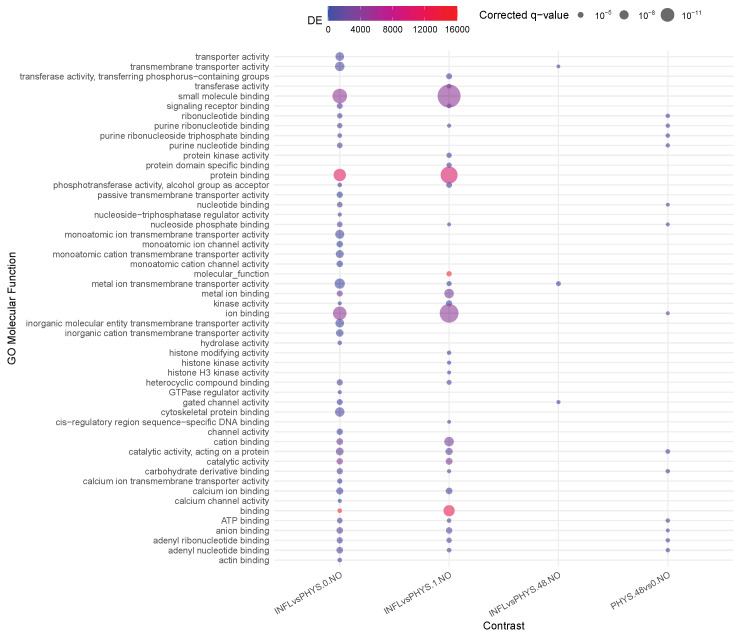
Enrichment analysis using gometh for the impact of time on physiological samples and inflamed samples.

Inflamed samples do not show statistically relevant changes, while PHYS samples present over time a decrease in *binding* (*ion, cation*) (Figure 1). The difference between PHYS and INFL at the methylomic level is represented by a reduced *transmembrane transporter activity*, stable over time and supported at earlier times by transient enhanced *ion binding* and *catalytic activity*, which is coherent with a more pronounced cell stress response in INFL versus PHYS and also supported by an increase in methylage over time, in which acceleration is more relevant in INFL.

### 3.2. Question 2: Impact of Stimuli on Physiological Samples

**Figure 2:**
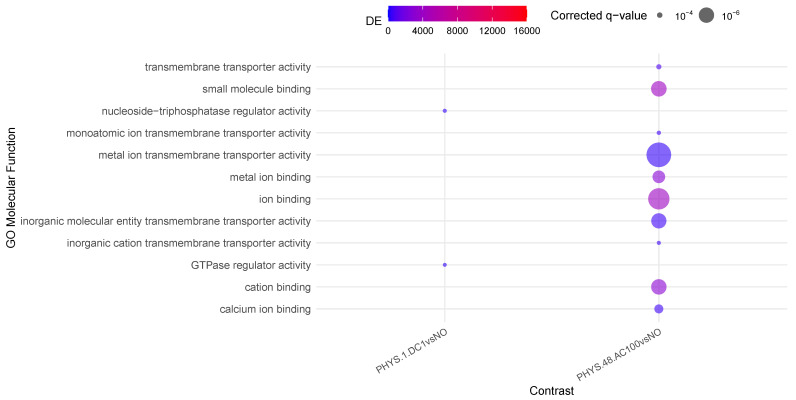
Enrichment analysis using gometh for the impact of stimulus on physiological samples.

In Figure 2, very little activity appears to involve differential methylation. In the steady state, only AC100 appears to strongly decrease *transmembrane transportation* and mildly increase *ion binding*. Transiently, for DC1, we observe a decrease in *GTPase regulator activity*. Recalling Table 3 within this question, DC5 appears to mildly increase mitotic age, supporting the idea that such stimuli on non-inflamed samples should be administered with caution.

### 3.3. Question 3: Effects of Stimuli on Inflamed Samples

**Figure 3:**
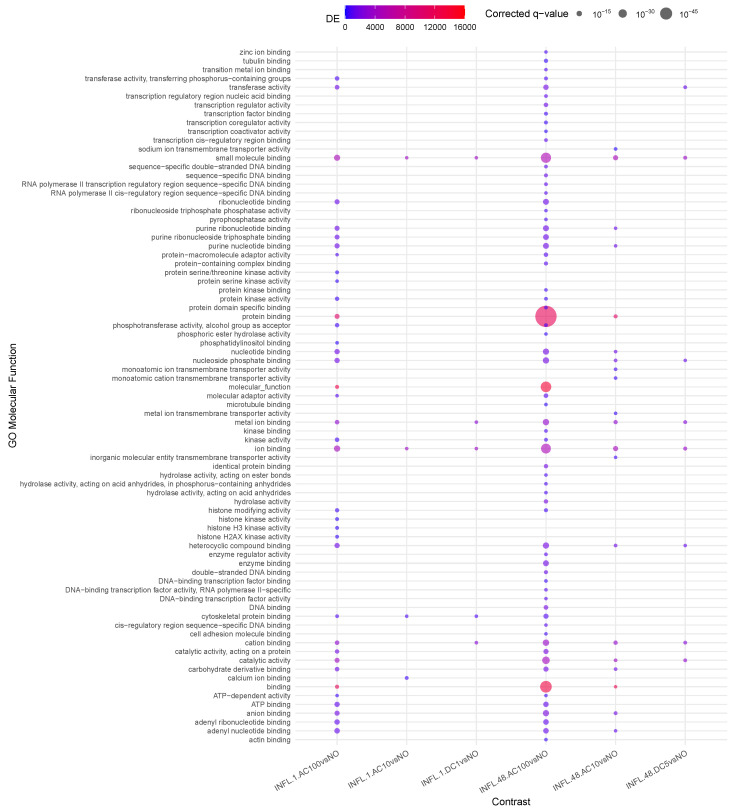
Enrichment analysis using gometh for the impact of stimulus on inflamed samples.

Considering the enriched functions (Figure 3), the major number of relevant contrasts refer to AC100, at all time points, with a significant enhancement of *protein binding activity* and a reduction in the *translational activity* at 48 h. Overall, the reduction in *translational activity* and *proliferation* supported at the transcriptomic and epigenomic levels possibly indicates a complex interaction of cellular stress responses, epigenetic modifications, shifts in gene expression patterns, potential differentiation cues, and changes in cellular communication. Together, these factors drive a transition from proliferation to maintenance and adaptation mechanisms under stress conditions. Methylation is a robust and long-lasting epigenetic modification, retaining a degree of plasticity, allowing it to respond to developmental and environmental factors over time. This could therefore imply a stable and hence durable effect of the observed phenomena triggered by AC100.

In AC10, some of the same *protein binding activity* is visible, although milder, suggesting a frequency-dependent intensity of the triggered activity and *transcription* is replaced by *transmembrane transporter activity*.

DC1 presents in transcriptomics early inflammatory activity followed by proliferation, echoing the phase of wound healing (epithelial–mesenchymal transition type 2, [15]), accompanied by a mild reduction in methylage. Together, these observations lead to the recommendation for further investigation into the potential for translational anti-inflammatory applications of this stimulus.

### 3.4. Question 4: Impact of States on Stimulus

**Figure 4:**
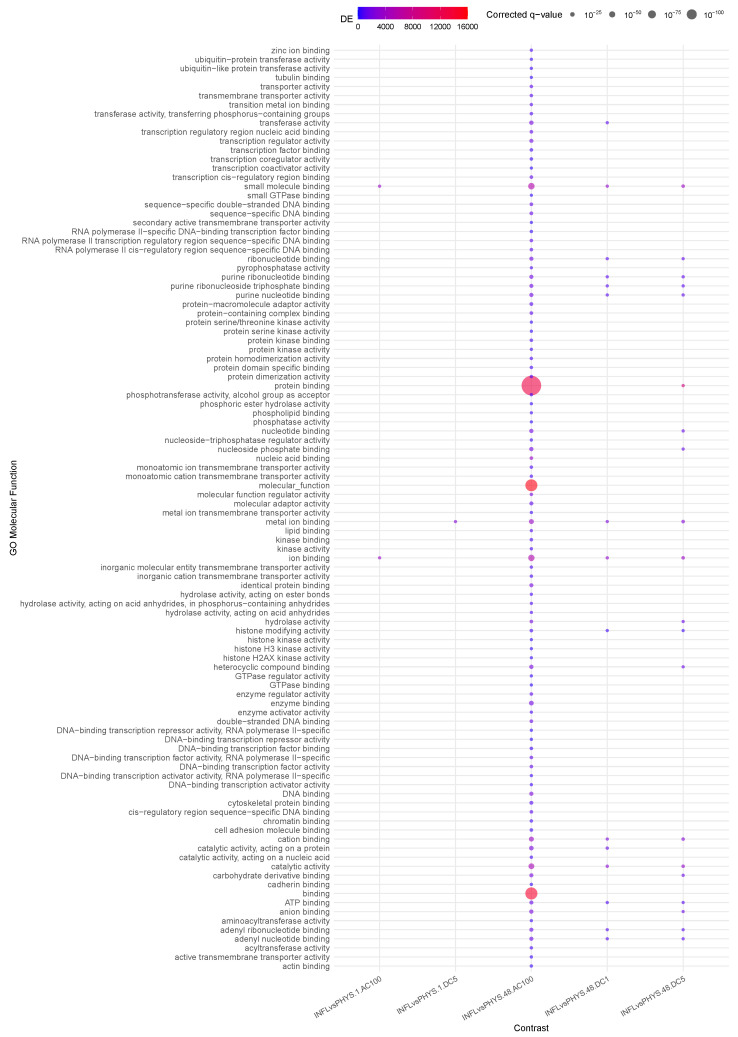
Enrichment analysis using gometh for the impact of states on stimuli.

This question aims to clarify the information gained in Questions 2 and 3 by directly assessing the differential activity occurring in PHYS versus INFL.

(Figure 4) shows how the differential effect of AC100 is striking and indicates how, in the long term (48 h), this stimulus preserves the already observed *transmembrane transporter activity* difference observed between INFL and PHYS in the absence of stimuli (Question 1). Furthermore, AC100 enhances *molecular function activation* and *binding*, the latter also being elicited at a milder level by DC1-5.

Conversely, no enrichment is visible for AC10, indicating that the differences between PHYS and INFL at 48 h in the absence of stimuli (reduced *transmembrane transporter activity*) are canceled out at the methylation level. In other words, this suggests that AC10 has the potential to restore this *transmembrane activity*.

### 3.5. Question 5: Differential Impact of Stimuli on PHYS

This question clarifies Question 2 regarding the differential effects of stimuli on PHYS samples.

**Figure 5:**
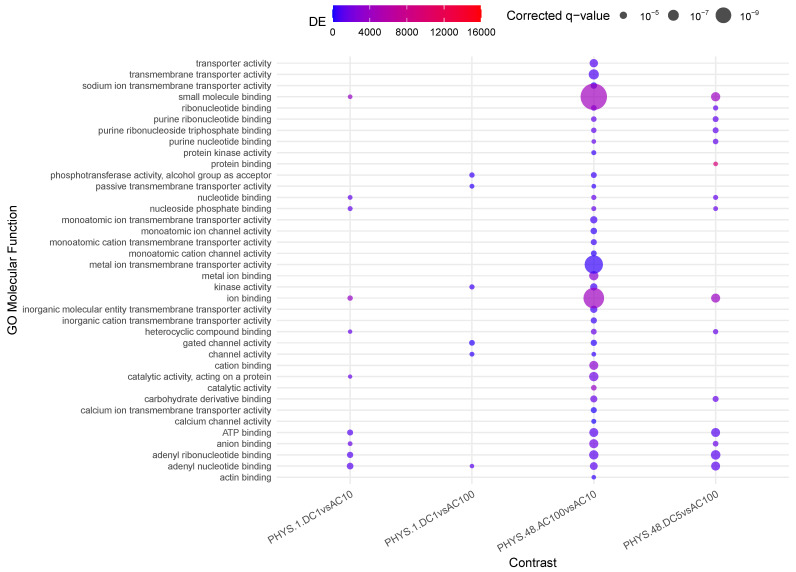
Enrichment analysis using gometh for the impact of stimuli on PHYS.

When looking at the plot in Figure 5, *PHYS.48.AC100vsAC10* and, to a minor extent, *PHYS.48.DC5vsAC100*, there is an increase in the *catalytic activity* in the cells and a reduction in *transmembrane transporter activity*. This activity appears to be graded, with AC100, then DC5 and AC10 slowly reducing their differential effects (in other words, DC5 is equivalent to AC10 on PHYS). DC1 appears to be involved only transiently (1 h) in methylation changes.

### 3.6. Question 6: Differential Impact of Stimuli on INFL

**Figure 6:**
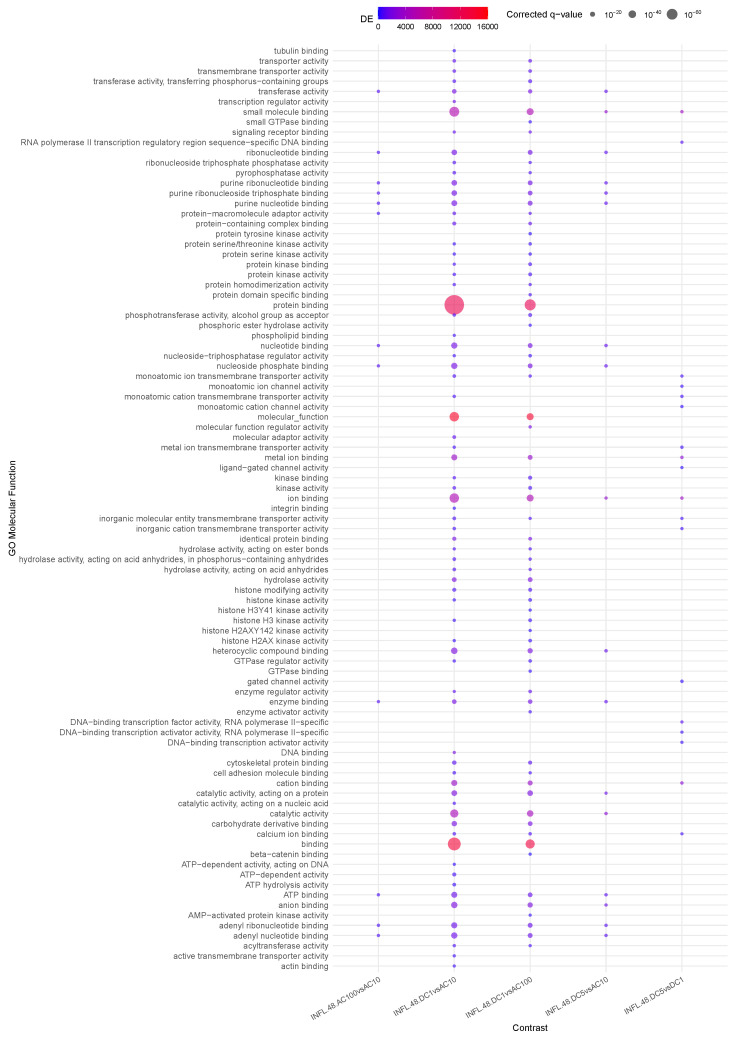
Enrichment analysis using gometh for the impact of stimuli on INFL.

Finally, Question 6, presented in Figure 6, deepens the observations of Question 3 on the effects of stimuli on INFL samples.

Early activity (1 h) appears to be non-differential, that is, all stimuli elicit the same type of activation or no early genes are involved, i.e., globally, no significant enrichment is observed for early samples. Conversely, in the long term (48 h), several differences are visible. From the observation drawn in Question 3, we recall that ACs are associated with an enhancement of *ion binding* (since this phenomenon is shared by AC10 and AC100, it is not significant here, where only differential activities are highlighted) and differential *transmembrane* vs. *translational activity* associated with AC10 and AC100, respectively. This difference translates here to a significant difference in *purine binding*, reduced in AC100 (*INFL.48.AC100vsAC10*).

Conversely, DC voltage impacts *metal ion binding* and *transmembrane transportation* (*INFL.48.DC5vsDC1*).

Finally, DC1 compared to any AC appears to elicit molecular function activity via *protein binding* and *transporter activity* and reduced *purine binding* more effectively at 10 Hz than 100 Hz, where we see a mildly reduced set of enriched functions.

This is even more pronounced at 5 V DC, which presents no differential activity when compared to AC100, and reduced *purine binding* in AC10 (*INFL.48.DC5vsAC10*). Overall *purine binding* appears to represent the core of the activity of AC10.

## 4. Conclusions

We have completed our first systematic exploration of the molecular landscape affected by ES in inflamed conditions. Despite the clear limits of our model and the number of stimuli, we determine from this study that ES is a versatile tool able to elicit a variety of effects that are time-, state- (inflamed or not), modulation- (direct or alternate current), and voltage intensity-dependent. While this offers additional and unprecedented information on the effects of ES, more detailed mechanistic insights in particular, regarding inflammation and senescence are needed to accelerate the potential for translation. Importantly, our result confirms that caution in the application of ES should be taken given the very diverse effects obtained. Finally, we wish to highlight that while the simplicity of our construct represents a limit in the translation of the result, it also clearly highlights how ES has an impact on basic and highly conserved mechanisms that are unrelated to the autonomic nervous system and which should be taken into consideration in bioelectronic medicine. We hope that this first overview will trigger more complete and systematic analyses to better elucidate the potential and limitations of such approaches for the greatest benefit of patients.

## Figures and Tables

**Table 1:** Study design. The first four rows represent the main variables explored in the study, namely inflammatory state (1st row); stimulus where NO stands for “no stimulus” (2nd row); further stimulus parameters (if any, 3rd row); and sampling time (4th row).

INFL											PHYS										
*NO*			DC				AC				*NO*			DC				AC			
			1 V		5 V		10 Hz		100 Hz					1 V		5 V		10 Hz		100 Hz	
t0	t1	t48	t1	t48	t1	t48	t1	t48	t1	t48	t0	t1	t48	t1	t48	t1	t48	t1	t48	t1	t48

**Table 2:** Experimental questions, associated contrasts, and summary results. Contrasts’ labels are constructed by the combination of the value of three variables (state, type of stimulus, time point) per condition compared, where vs. serves as a separator of the differential condition. The column titled RNA and Metabolites summarizes the results from [5], and column mDNA and Methylage shows the current results. ^†^ indicates statistically significant changes that however did not survive correction for multiple hypotheses. BCAA stands for branched chain ammino acids.

Question	Contrasts	RNA & Metabolites	mDNA & Methylage
1. What is the impact of time?	PHYS.1vs0.NO, PHYS.48vs1.NO, PHYS.48vs0.NO, INFL.1vs0.NO, INFL.48vs1.NO, INFL.48vs0.NO, INFLvsPHYS.0.NO, INFLvsPHYS.1.NO, INFLvsPHYS.48.NO	Reduced proliferation and BCAA metabolism Increased hypoxia (PHYS) Reduced inflammation (INFL) Senescent trend over time (more so in INFL)	Reduced *transmembrane transporter activity* (INFL vs. PHYS) No change over time (INFL) Decrease in *ion binding* (PHYS) Increased methylage trend (INFL vs. PHYS)
2. What is the impact of stimuli on the physiological state?	PHYS.1.DC1vsNO, PHYS.1.DC5vsNO, PHYS.1.AC10vsNO, PHYS.1.AC100vsNO, PHYS.48.DC1vsNO, PHYS.48.DC5vsNO, PHYS.48.AC10vsNO, PHYS.48.AC100vsNO	DC1 is inflammatory, with senescence pattern AC100 has reduced interferons and energy production and BCAA metabolism DC5 shows *transitional* activity AC10 has reduced proliferation, senescence pattern	DC1 transiently decreases *GTPase regulation* AC100 decreases *transmembrane transportation* DC5, mild ^†^ increase in methylage
3. What is the impact of stimuli on the inflamed state?	INFL.1.DC1vsNO, INFL.1.DC5vsNO, INFL.1.AC10vsNO, INFL.1.AC100vsNO, INFL.48.DC1vsNO, INFL.48.DC5vsNO, INFL.48.AC10vsNO, INFL.48.AC100vsNO	DC5 epithelial–mesenchymal transition AC100 has reduced proliferation AC10 and DC1 show early inflammation followed by proliferation (no decrease in inflammation)	DC5 reduced *ion transferase* and *catalytic activities* AC100 enhancement of *ion binding* Reduction of *translational activity* AC10 Enhancement of *ion binding* reduction in *transmembrane transporter activity* DC1 mild ^†^ decrease in methylage
4. What is the impact of states on stimulus?	INFLvsPHYS.1.DC1, INFLvsPHYS.1.DC5, INFLvsPHYS.1.AC10, INFLvsPHYS.1.AC100, INFLvsPHYS.48.DC1, INFLvsPHYS.48.DC5, INFLvsPHYS.48.AC10, INFLvsPHYS.48.AC100	ACs is pro-inflammatory with enhanced proliferation and frequency-dependent bile acids vs. oxidative phosphorylation activity (10 Hz vs. 100 Hz) (PHYS) ACs is mildly anti-inflammatory (INFL) DCs is anti-inflammatory with enhanced proliferation (stronger in DC5 than AC100)	AC10 cancel reduced *transmembrane transport* of INFL AC100 maintain *transmembrane transport* difference in INFL vs. PHYS but enhances *binding*, *molecular function activity* DC1-5 mildly enhances binding
5. What is the differential impact of stimuli on PHYS?	PHYS.1.DC5vsDC1, PHYS.48.DC5vsDC1, PHYS.1.AC100vsAC10, PHYS.48.AC100vsAC10, PHYS.1.DC5vsAC10, PHYS.1.DC5vsAC100, PHYS.48.DC5vsAC10, PHYS.48.DC5vsAC100, PHYS.1.DC1vsAC10, PHYS.1.DC1vsAC100, PHYS.48.DC1vsAC10, PHYS.48.DC1vsAC100	DC5 and AC10 have no relevant impact on inflammation, contrasting proliferative activity (mildly down for AC10 and mildly up for DC5) AC100 is mildly anti-inflammatory DC1 is mildly pro-inflammatory	DC5 and AC10 are equivalent AC100,DC5,AC10 progressively increase *catalytic activity* and reduce *transmembrane transporter activity* associated with mild ^†^ increase in methylage
6. What is the differential impact of stimuli on INFL?	INFL.1.DC5vsDC1, INFL.48.DC5vsDC1, INFL.1.AC100vsAC10, INFL.48.AC100vsAC10, INFL.1.DC5vsAC10, INFL.1.DC5vsAC100, INFL.48.DC5vsAC10, INFL.48.DC5vsAC100, INFL.1.DC1vsAC10, INFL.1.DC1vsAC100, INFL.48.DC1vsAC10, INFL.48.DC1vsAC100	DC1 and AC10 do not show significant lasting (48 h) enrichment, indicating the same type of effect DC1 and AC10 more proliferative than AC100 AC100 has lower proliferation and energy production (compared to baseline) DC5 is anti-inflammatory and proliferative vs. AC100	Early (1 h) activity shared or non-existent (no differential results) AC10 elicits *purine binding* (it is reduced in all other stimuli when compared) DC1 vs. ACs enhances *molecular function activity* via *non-covalent protein binding* and *transporter activity* DC differences in voltage affect *metal ion binding* and *transmembrane transportation*

**Table 3:** Results of the EpiTOC analysis, with significant enrichment (✓) for functions involved in senescent-like activity in transcriptomics (column RNA); significant results in EpiTOC (column DNAm); differences in average methylation (column Δm); *t*-statistics; and *p*-values for significant contrasts, with single (*p*-value) and Bonferroni–Hochberg corrected values (BH *p*-value).

Contrast	RNA	DNAm	Δm	t	*p*-Value	BH *p*-Value
INFLvsPHYS.48.NO	✓	✓	0.0181	10.385	0.000883	0.0503
PHYS.48.DC1vsNO	✓	-	0.0081	3.1856	0.0577	0.4111
PHYS.48.AC10vsNO	✓	-	0.00565	2.6269	0.0784	0.4965
PHYS.48.DC5vsNO	-	✓	0.00658	4.0624	0.01736	0.9895
INFLvsPHYS.48.DC1	-	✓	−0.012	−4.1344	0.0041	0.2341
INFL.48.DC1vsNO	-	✓	−0.01356	−9.429	0.01106	0.6305

## Data Availability

Methylomic data are available at Gene Expression Omnibus with ID GSE280243.

## Supplementary Materials

The following supporting information can be downloaded at: https://www.mdpi.com/article/10.3390/cells14110838/s1, Table S1: Methylage, Tables S2–S7: Differentially methylated CpG (grouped by questions); Supplementary Materials file containing a schematic representation of the whole work pipeline and details on the experimental methods for the 3D bioconstruct. Supporting information.pdf includes a graphical representation of the analysis (Figure S1) and details on the 3D bioconstruct preparation.
