# Supplementary material for: Modified Methylation Following Electrostimulation in a Standardized Setting—Complementing a Transcriptomic Analysis
Source: Cells. 2025 Jun 4;14(11):838. doi: 10.3390/cells14110838 (PMC12155531; doi:10.3390/cells14110838)
Supplement: Supplementary file 1 [file cells-14-00838-s001.zip › Supporting information.pdf]

Supporting Information

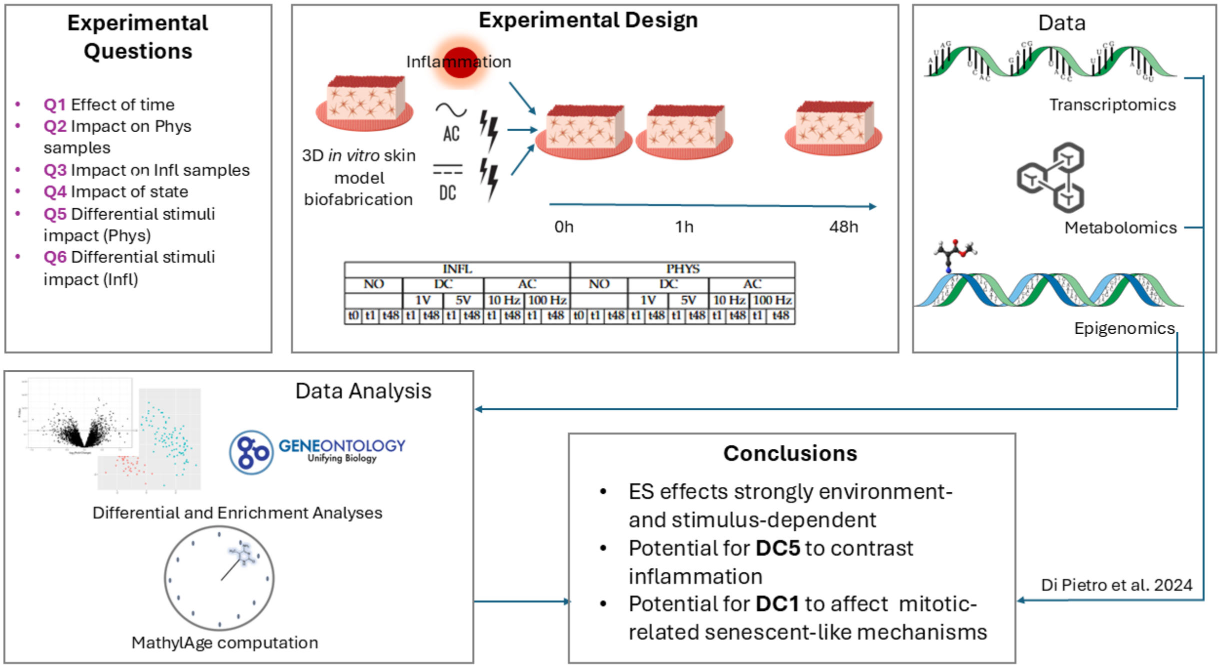

Figure S1 Scheme representing the analyses run in this work and the previous accompanying paper. The experimental question motivating the work (I.e. the potential anti-inflammatory effects of electrical stimuli) was broken into six subquestions to be addressed by dedicated experiments. The experimental setup was designed to maximize reproducibility (3D bioconstruct) and enable results comparisons, exploring differential stimuli types (direct and alternate current) and magnitude (1V, 5V, 10Hz and 100Hz), as well as different physiological and inflamed states. Omics were performed on the samples to explore the transcriptional and metabolic levels (Di Pietro et al., 2024) as well as the methylomic and methylage. Integration of the results recommends further exploration of the DC stimuli on inflamed samples.

In vitro skin model biofabrication protocol

To obtain each 3D in vitro skin model, 350 µL of type 1 rat-tail collagen (Roche, Basel, Switzerland) dissolved in acetic acid 0.2%v/v was supplemented with 15 µL of sterile ddH2O, 35 µL of NaOH 0.5 M, 50 µL HEPES 0.2 M, 50 µL DMEM 10X (Sigma Aldrich, St. Louis, MO, USA) and 25 µL of human fibroblasts (HFF-1, ATCC). Fibroblasts were at a final concentration of 1.5×10^6 cells/ml of mixture. 500 µL of HFF-1-laden collagen was seeded

onto PET 12-well hanging inserts with a porosity of 0.4 μm (Milicell®, Merck, Rahway, NJ, USA). Samples were incubated for 30 min at 37 ◦C to let the collagen crosslink. Once the stable dermal layer was established, 2 × 10^5 human keratinocytes (HaCaT, Antibody Research Corporation, St. Charles, MO, USA) in 500 μL of complete DMEM were seeded on top of the dermis compartment. After three days of submerged culture in complete DMEM (15% FBS, 2% L-glutamine, 1% penicillin-streptomycin, 1% sodium pyruvate) to facilitate keratinocyte adhesion to the dermis, Air-Liquid Interface (ALI) culture started providing 400 μL of 3dGROTM Skin Differentiation Medium (Sigma Aldrich, St. Louis, MO, USA) in the lower compartment while leaving the upper compartment exposed to air. The culture was maintained for a total of fourteen days.
