# Supplementary figures and images for: Modified Methylation Following Electrostimulation in a Standardized Setting—Complementing a Transcriptomic Analysis
Source: Cells. 2025 Jun 4;14(11):838. doi: 10.3390/cells14110838 (PMC12155531; doi:10.3390/cells14110838)

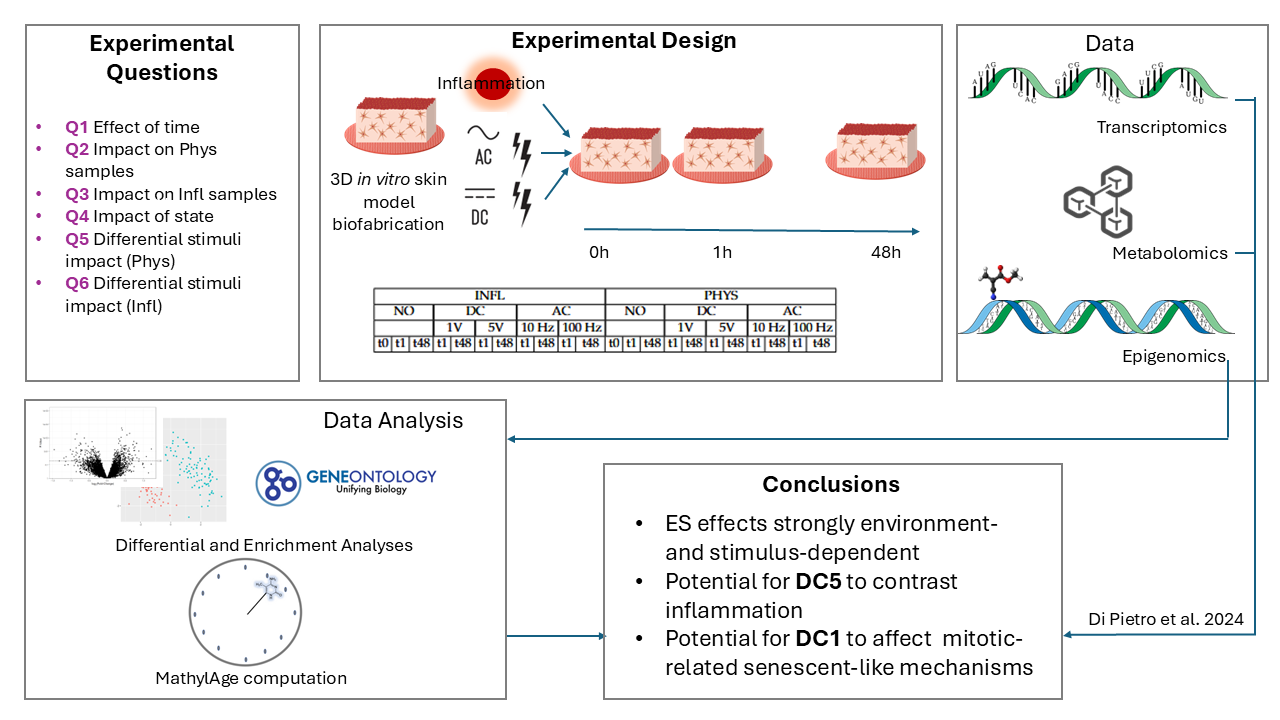

Supplement: Supplementary file 1 [file cells-14-00838-s001.zip › FigS1.tif]
